# Interruption after Short-Term Nitrogen Additions Improves Ecological Stability of *Larix olgensis* Forest Soil by Affecting Bacterial Communities

**DOI:** 10.3390/microorganisms12050969

**Published:** 2024-05-11

**Authors:** Tongbao Qu, Xiaoting Zhao, Siyu Yan, Yushan Liu, Muhammad Jamal Ameer, Lei Zhao

**Affiliations:** College of Forestry and Grassland, Jilin Agricultural University, Changchun 130118, China; qvtb@jlau.edu.cn (T.Q.); 13072255117@163.com (X.Z.); 16643179269@163.com (S.Y.); 18943478100@163.com (Y.L.); jameer6524@gmail.com (M.J.A.)

**Keywords:** nitrogen addition, interruption, bacterial community, bacterial diversity, soil ecological health

## Abstract

Atmospheric nitrogen deposition can alter soil microbial communities and further impact the structure and function of forest ecosystems. However, most studies are focused on positive or negative effects after nitrogen addition, and few studies pay attention to its interruption. In order to investigate whether interruption after different levels of short-term N additions still benefit soil health, we conducted a 2-year interruption after a 4-year short-term nitrogen addition (10 and 20 kg N·hm^−2^·yr^−1^) experiment; then, we compared soil microbial diversity and structure and analyzed soil physicochemical properties and their correlations before and after the interruption in *Larix olgensis* forest soil in northeast China. The results showed that soil ecological stabilization of *Larix olgensis* forest further improved after the interruption compared to pre-interruption. The TN, C:P, N:P, and C:N:P ratios increased significantly regardless of the previous nitrogen addition concentration, and soil nutrient cycling was further promoted. The relative abundance of the original beneficial microbial taxa *Gemmatimonas*, *Sphingomonas*, and *Pseudolabrys* increased; new beneficial bacteria *Ellin6067*, *Massilia*, *Solirubrobacter*, and *Bradyrhizobium* appeared, and the species of beneficial soil microorganisms were further improved. The results of this study elucidated the dynamics of the bacterial community before and after the interruption of short-term nitrogen addition and could provide data support and a reference basis for forest ecosystem restoration strategies and management under the background of global nitrogen deposition.

## 1. Introduction

The extensive burning of fossil fuels and the use of fertilizers have increased the rate of nitrogen deposition and affected the structure and function of ecosystems [1,2,3,4,5]. The forest ecosystem, as the most important ecosystem on land, is directly affected by atmospheric nitrogen deposition. Moderate N additions can promote plant and microbial growth by altering soil nutrient cycling [6]. However, when the nitrogen content in the ecosystem reaches saturation, excessive nitrogen addition may lead to soil nutrient imbalance, inhibit plant growth, and alter microbial structure and function, further reducing ecosystem stability [3,7,8]. Most studies tend to focus on the ecological effects of soil microorganisms and the continuous effects of N addition, but there are few studies on the effects of interruption after short-term N additions [9,10,11]. Thus, further investigation on the dynamics of microbial community structure and diversity after interruption of short-term N additions can provide a theoretical basis for the sustained effects of increased nitrogen deposition on nutrient cycling and soil health in forest ecosystems.

Microorganisms drive the biogeochemical cycles in soil and are key drivers and regulators of forest ecosystem productivity and diversity [10,12]. Compared with fungi, bacteria have small size, rapid reproduction and renewal, strong metabolic ability, and high sensitivity to changes in foreign substances, so they are more responsive to changes in the external environment [13]. Previous studies have recognized that N addition affects the structure, composition, and diversity of microbial communities, resulting in positive, negative, or no-impact outcomes [14,15]. Most of these studies have confirmed that N addition alters soil nutrition condition and accelerates soil acidification, thereby reducing the diversity of soil microorganisms, and ultimately destroys the ecosystem function of the forest ecosystem [16,17]. Moreover, some nitrogen remains in the soil for a longer time after long-term N additions are interrupted, and the negative effects of N additions on the ecosystems continue [18,19]. However, in nitrogen-limited temperate forests, N addition has alleviated nitrogen limitation and stimulated microbial activity, which benefits ecosystem health [20,21]. N addition reduces competitive effects between bacterial taxa by increasing soil nutrient content, allowing more microorganisms to maintain symbiotic relationships, thereby improving functional diversity and structural stability [22]. 

The *Larix olgensis* forest is one of the main afforestation species in northeast China because of its strong adaptability, fast growth, and high economic and ecological values. However, northeast China is severely affected by N deposition [23], and due to poor biodiversity and monoculture, the productivity of the *Larix olgensis* plantation forest is relatively low [24]. Previous studies have shown that short-term N additions are significantly positive for *Larix olgensis* forests, but the effect of interruption after short-term N addition on *Larix olgensis* forests has not been studied [25]. Based on the above understanding, we conducted a two-year interruption experiment after short-term N additions of different levels in a *Larix olgensis* forest; the soil nutrient diversity and soil microbial community structure were measured, respectively, and the effects of interruption on soil ecosystem were explored by comparison. We hypothesized that soil interruption after short-term N addition in *Larix olgensis* forests has a negative effect on soil health by altering bacterial population structure and diversity. Our research explains the dynamic response of soil health condition to interruption and supports a reference for better understanding the mechanisms of N additions in *Larix olgensis* ecosystems and the sustainable management of plantation forests.

## 2. Materials and Methods 

### 2.1. Experimental Area and Design

This research was conducted in a *Larix olgensis* forest, which is located in the campus of Jilin Agricultural University (43°05′–45°15′ N; 124°18′–127°05′ E), Changchun, Jilin Province, China. Changchun has a monsoon climate of medium latitudes, with a mean annual temperature of 4.8 °C and a mean annual precipitation of 570.3 mm. The months with the highest precipitation in the whole year are from July to August. There is a scarcity of species in the understory, with an average coverage ranging from 2 to 4%, dominated by *Larix olgensis*, *Bromus inermis*, *Chelidonium majus*, and *Viola prionantha.*

The interruption test started in 2021 and stopped in 2023. The N addition test before interruption was as follows: The experiment (from 2018 to 2021) was designed with sample sites; three N treatments were set, including control (CK: 0 kg N·hm^−2^·yr^−1^), low nitrogen (LN: 10 kg N·hm^−2^·yr^−1^), and high nitrogen (HN: 20 kg N·hm^−2^·yr^−1^), and NaNO_3_ nitrogen fertilizer was applied. A total of 15 sample plots of 5 × 5 m were established, with at least a 2 m wide buffer zone between each site to avoid interference between sample plots. There were three treatments in the experiment, with similar site and stand characteristics, and each treatment had five replicates. N addition treatments were conducted in May and October each year by mixing N with 1 kg of sand (sand passed through a sieve and impurities removed) and applying it to the soil surface. From August 2021, all sites were not treated with N addition to maintain natural recovery.

The soil samples were collected in August, of which 2021 was sampled after a 4-year short-term N addition, and 2023 was sampled after a 2-year interruption. The humus layer on the soil surface was removed before sampling to expose the soil. There were 5 sampling locations in each experimental plot, and the soil samples were taken by 5 cm soil auger within the 0–10 cm soil layer. Five soil samples were blended into one sample at each site using the five-point sampling method, placed in self-sealing bags, and preserved by dry ice and shipped back to the laboratory. In total, 30 composite samples were obtained during this study. Each fresh sample was separated into two portions after screening out soil impurities by a 2 mm sieve. One portion of the samples was stored in a refrigerator at −80 °C for the determination of microbial community structure and diversity, and another portion of the samples was sieved in an air-dried state for the determination of soil water content (SWC), soil pH, soil total carbon (TC), soil total nitrogen (TN), and soil total phosphorus (TP).

### 2.2. Soil Index Determination Method

#### 2.2.1. Chemical Test Methods of Soil Properties

Determination of pH: the soil pH was measured by potentiometry by putting 10 g of the air-dried soil sample sieved through a 2 mm sieve into a 50 mL beaker and adding 25 mL of distilled water (1:2.5 soil/water ratio). The pH value of the soil sample was determined using a pH meter after thorough mixing with a glass rod and standing for 2 h (PHS-25, Shanghai, China).

Determination of SWC: the SWC was measured by oven-drying method by putting 10 g of fresh soil samples screened through a 2 mm sieve in a tinfoil shell and drying in a thermostat at 105 °C for 24 h to determine the SWC [26].

Determination of soil TC, TN, and TP: TC content was measured with the H_2_SO_4_-K_2_Cr_2_O_7_ method. TN content was measured by the semi-Kjeldahl method. After digesting soil samples with H_2_SO_4_ and HClO_4_, TP content was measured by molybdenum–antimony colorimetry [27]. 

#### 2.2.2. Determination of Soil Microbial Community Structure

The genomic DNA of soil samples was extracted by the CTAB or SDS method, which was repeated 3 times. The purity and concentration of the DNA were detected by electrophoresis on agarose gels, and after obtaining the results, the samples were diluted to 1 ng/µL with a centrifuge tube and sterile water.

DNA fragments covering the bacterial 16S V4 region were amplified, and bacterial libraries were constructed to characterize bacterial diversity. The specific primer barcodes were 515F and 806R. All PCR reactions were performed using 0.2 µM of forward and reverse primers and approximately 10 ng of template DNA for a total of 30 thermal cycles. The amplification program consisted of an initial denaturation at 98 °C for 1 min, followed by 10 s at 98 °C, 30 s at 50 °C, 30 s at 72 °C, and a final extension at 72 °C for 5 min for cooling. This was performed in 15 µL Phusion^®^ High—Fidelity PCR Master Mix (New England Biolabs, Beverly, MA, USA).

Eligible PCR products were purified by magnetic bead digestion, and aliquots of the samples were mixed using the PCR product concentration as a reference. The PCR products were detected by electrophoresis on a 2% agarose gel, and the target bands were recovered. Sequencing libraries were indexed and quantified using Qubit and Q-PCR. PCR amplicon sequencing was performed on an Illumina NovaSeq 6000 sequencer (Illumina, San Diego, CA, USA). The above sequencing was commissioned to Tianjin Novozymes Biotechnology Co, Tianjin, China.

### 2.3. Statistical Analysis

Data statistics and analysis were carried out with SPSS 26.0. Graphs were drawn with GraphPad Prism 9 and Origin2021 software. The effects of interruption after short-term N additions on soil physical and chemical properties and bacterial diversity in *Larix olgensis* forests were evaluated by one-way ANOVA and repeated-measures ANOVA. Multiple comparisons of the significance of differences between treatments were performed using the LSD method (*p* < 0.05). The correlation analysis after interruption of soil environmental factors and bacterial diversity was performed by Pearson correlation analysis. Using soil bacterial dominant taxa as samples and environmental factors as variables, Canoco 5.0 redundancy analysis was employed to investigate the main factors influencing soil bacterial diversity.

## 3. Results 

### 3.1. Effects of Interruption after Short-Term Nitrogen Additions on Soil Properties

**Figure 1:**
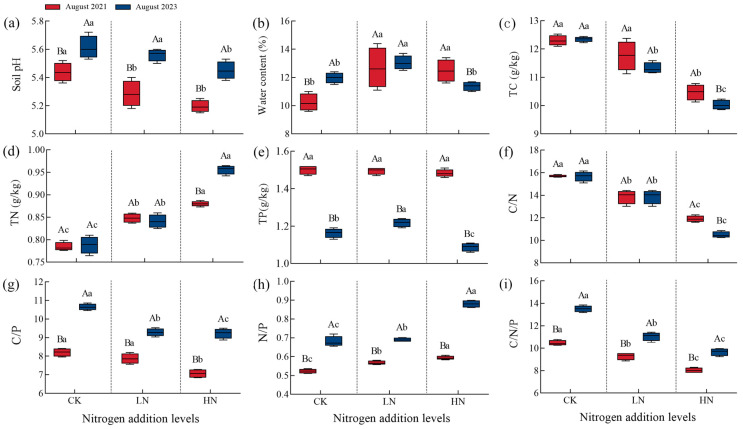
Effect of interruption after short-term N additions on soil environmental factors. CK: 0 kg N hm^−2^·yr^−1^, LN: 10 kg N hm^−2^·yr^−1^, HN: 20 kg N hm^−2^·yr^−1^; (**a**): soil pH, (**b**): SWC, (**c**): TC content, (**d**): TN content, (**e**): TP content, (**f**): C:N value, (**g**): C:P value, (**h**): N:P value, (**i**): C:N:P value. Different lowercase letters indicate differences between high and low N additions in the same year (*p* < 0.05), Different capital letters indicate differences between 2021 and 2023 at the same N addition (*p* < 0.05).

As shown in Table 1 and Figure 1, the results show that after interruption of short-term N additions, soil pH and TN content showed a significant increase with increasing levels of previous N addition (*p* < 0.01), while TC content showed a significant decrease (*p* < 0.01). In addition, the interruption also significantly affected SWC (*p* < 0.01). Compared to pre-interruption, the trend in soil pH, SWC, TN, and TC content was basically the same. We found that high N additions significantly increased TN by 8.6%, and TC decreased by 4.3% (*p* < 0.01). Regardless of the level of previous nitrogen, soil pH in 2023 was significantly greater than in 2021 by 5.2% and 4.9% (*p* < 0.01).

Our research indicated that after interruption of short-term N additions, soil C:N, C:P, and C:N:P ratios indicated a significant decreasing trend with increasing levels of previous N addition, while soil N:P was the opposite. In addition, the interruption of short-term N additions also significantly affected TP content (*p* < 0.01). Compared to pre-interruption, the trends of the indicators in 2023 were generally consistent, and the effect on the N:P ratio showed a gradual trend with the level of previous N addition, while the C:N and C:N:P ratios showed the opposite trend. We found two levels of previous N addition significantly increased the C:P ratio by 18% and 30.6%; N:P ratio by 21.6% and 48.2%; and C:N:P ratio by 19.1% and 20.3%. Furthermore, TP content under the low and high N additions was significantly lower than those of 2021 by 18.4% and 26.7%, respectively; the C:N ratio was similarly significantly decreased by 2.9% and 11.8%. 

### 3.2. Effect of Interruption after Short-Term Nitrogen Additions on Soil Bacterial Diversity

**Figure 2:**
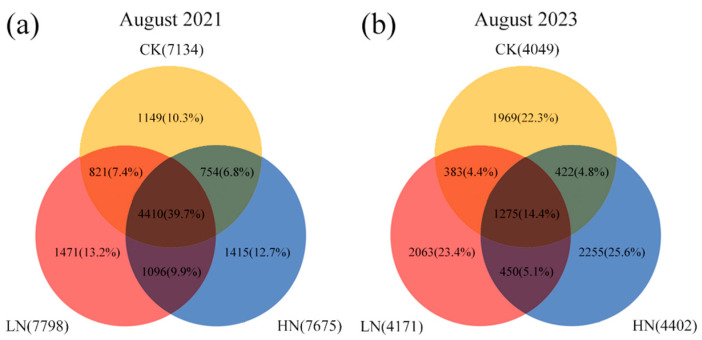
Venn plots of interruption after short-term N additions on soil bacteria. CK: 0 kg N hm^−2^·yr^−1^, LN: 10 kg N hm^−2^·yr^−1^, HN: 20 kg N hm^−2^·yr^−1^, (**a**): August 2021, (**b**): August 2023, Yellow: control treatment, Red: low-nitrogen treatment, Blue: high-nitrogen treatment.

The number of shared and unique OTUs across treatments in the Venn diagram visualized the specificity and similarity of OTUs’ composition before and after the interruption of short-term N additions. As shown in Figure 2, compared to pre-interruption, the number of OTUs reduced in every treatment after interruption, with the greatest decrease in the previous low-nitrogen treatment. The number and proportion of shared OTUs between treatments decreased, with the largest decrease observed in the number of shared OTUs among the three treatments. However, the number and proportion of OTUs unique to each treatment increased, with the largest increase in the number of OTUs unique to the previous high-nitrogen treatment.

**Figure 3:**
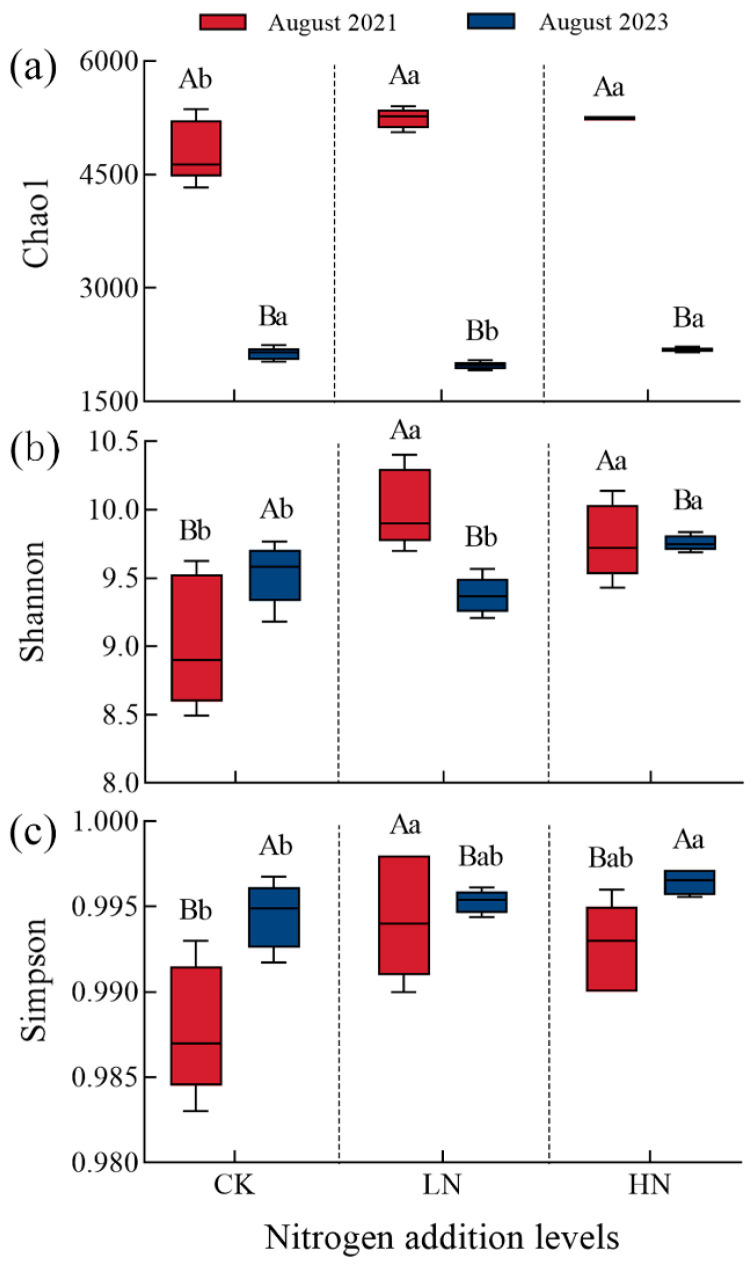
Effect of interruption after short-term N additions on bacterial diversity indices. CK: 0 kg N hm^−2^·yr^−1^, LN: 10 kg N hm^−2^·yr^−1^, HN: 20 kg N hm^−2^·yr^−1^; (**a**): Chao1 index, (**b**): Shannon index, (**c**): Simpson index. Different lowercase letters indicate differences between high and low N additions in the same year (*p* < 0.05), Different capital letters indicate differences between 2021 and 2023 at the same N addition (*p* < 0.05).

Interruption after short-term N additions and previous N additions significantly affected the Chao1, Shannon, and Simpson indices (*p* < 0.05). The interaction between N addition and interruption was significant for Chao1 and Shannon indices (*p* < 0.01), but not for Simpson index (Figure 3). Compared to the control group, the overall increase range showed that previous high N addition > previous low N addition in 2023. This trend was exactly the opposite of 2021. We also found that Chao1 index under two levels of previous N addition was significantly reduced by 62.3% and 58.4% in 2023 compared to 2021 (*p* < 0.01), respectively, but Shannon and Simpson indices improved, although neither was significant. The impact of previous high N addition on microbial diversity was more significant than previous low N addition after interruption.

### 3.3. Effect of Interruption after Short-Term Nitrogen Additions on Soil Bacterial Structure

**Figure 4:**
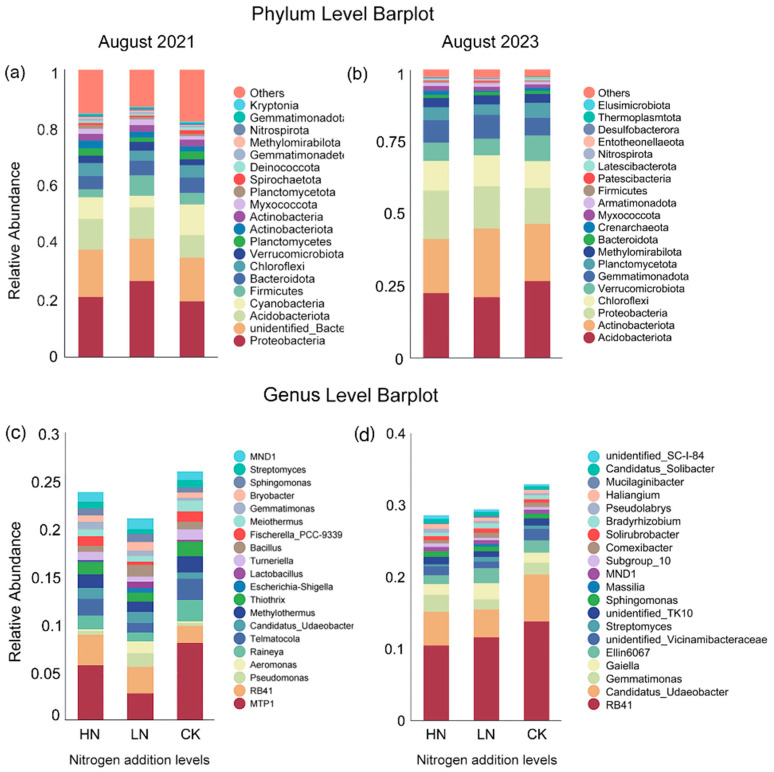
Comparison of short-term nitrogen additions and interruption on the abundance of bacterial. (**a**,**b**) Bacteria phylum level; (**c**,**d**) Bacteria genus level. Note: “Others” indicates the sum of the relative abundance of all gates except the top 20. CK: 0 kg N hm^−2^·yr^−1^, LN: 10 kg N hm^−2^·yr^−1^, HN: 20 kg N hm^−2^·yr^−1^.

Based on the OUT classification and taxonomic status, high-throughput sequencing analysis indicated a total of ten phylums with average relative abundance of bacterial phylum greater than 1% in all treated soil samples, which accounted for 93.2–94.1% of the total bacterial abundance after the interruption of short-term N additions (Figure 4), including *Acidobacteriota* (21.4–27.2%), *Actinobacteriota* (8.9–23.8%), *Proteobacteria* (12.5–16.7%), *Chloroflexi* (9.3–1.7%), *Verrucomicrobiota* (5.7–8.9%), *Gemmatimonadota* (6.1–8.2%), *Planctomycetota* (3.6–5.3%), *Methylomirabilota* (3.0–3.3%), *Myxococcota* (1.2–1.6%), and *Armatimonadota* (1.1–1.2%).

The effects of two previous N addition levels on the community structure of soil bacterial dominant bacteria were different. The specific performance was as follows: N addition increased the relative abundance of *Proteobacteria*, *Chloroflexi*, *Gemmatimonadota*, *Methylomirabilota*, *Myxococcota*, and *Armatimonadota*, decreased the relative abundance of *Acidobacteriota*, *Verrucomicrobiota*, and *Planctomycetota*. The impact on the relative abundance of *Actinobacteriota* showed a trend of low promotion and high inhibition. 

### 3.4. Relationships between Interruption Microbial Communities and Soil Properties after Short-Term Nitrogen Additions

We extracted the top 20 genera with average relative abundance to further analyze the effects of different levels of N additions on bacterial community structure at the genus level (Figure 4). We found that two previous N addition levels increased the relative abundance of *Sphingomonas*, *Solirubrobacter*, *Haliangium*, *Mucilaginibacter*, *Candidatus_Solibacte*; except *Solirubrobacter* and *Mucilaginibacter*, the relative abundance of the other three genera increased and was positively correlated with N additions’ levels. The impact of N addition on the relative abundance of the six genera *Gaiella*, *Ellin6067*, *Streptomyces*, *Massilia*, *Conexibacter*, *Bradyrhizobium* showed a trend of low promotion and high inhibition. The relative abundance of *Candidatus_Udaeobacter* was decreased.

**Figure 5:**
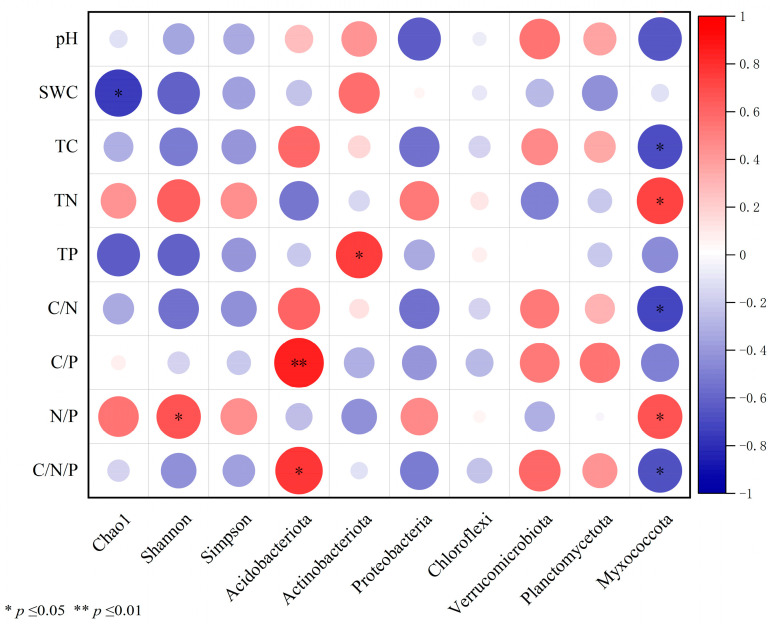
Correlation heat map of soil environmental factors and soil microbial community structure. * indicates significant correlation at the 0.05 level, ** indicates high correlation at the 0.01 level.

**Figure 6:**
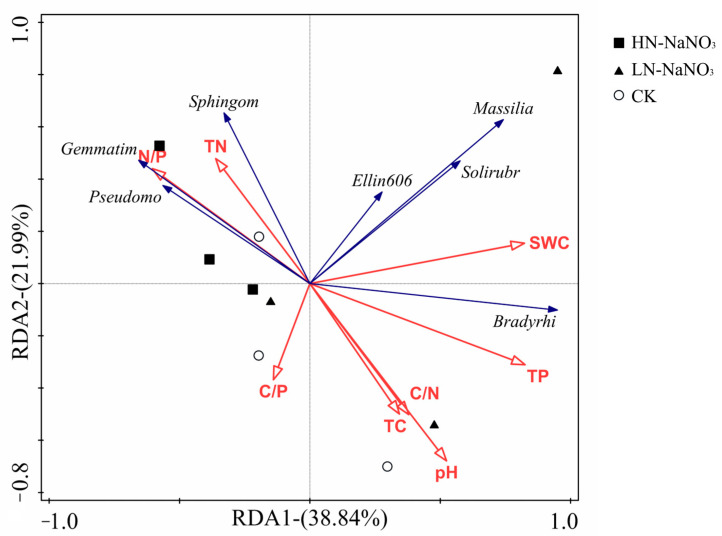
Redundancy analysis (RDA) of diverse environmental influences associated with dominant taxa of soil bacteria under interruption after short-term nitrogen additions.

According to the correlation analysis and redundancy analysis, the soil nutrient content and stoichiometric ratio were significantly correlated with beneficial microbiota. The main environmental factors affecting changes in the bacterial community structure were SWC and TP (Figure 5 and Figure 6). The above results indicated that even after interruption, previous short-term N additions could still further affect soil microbial communities by altering the soil N condition.

## 4. Discussion

### 4.1. Changes in Soil Properties before and after Interruption of Short-Term Nitrogen Additions

The N additions increased TN content by influencing external nitrogen input; NH_4_^+^ and NO_3_^−^ in soil also increased, which further aggravated the acidification of NH_4_^+^ and leaching of NO_3_^−^, eventually leading to soil acidification [28,29]. After interrupting short-term N addition, the pH value of the soil under the *L. olgensis* forest rebounded but remained acidic, suggesting that soil acidification caused by N addition continued after interrupting. Compared with 2021, the TN content in soil increased significantly with the previous addition of high N levels. In the experiment interrupted after a 10-year continuous N addition, TN content was also significantly higher than before the interruption, which is consistent with our findings [18]. This result may be attributed to soil nitrogen limitation being relieved and further increasing soil nitrogen supply capacity [25]. The consistent trend before and after the interruption of short-term N additions has indicated that the positive effects of previous N addition on soil ecology may persist, and these effects may be even more pronounced under conditions of high-level N addition.

The trend of TC content after interruption was the same as that in 2021, namely, the TC content decreased significantly with increasing N application. However, most studies have shown that short-term high N addition usually reduces TC content, which is inconsistent with our findings [26,30]. The reasons for this difference can be explained from the following three aspects. First of all, the N addition reduces the activity of most soil carbon cycle enzymes and reduces soil biological activity, resulting in lower rates of organic matter mineralization, which may lead to lower rates of decomposition and conversion of plant litter, and ultimately affecting soil carbon inputs [27,31,32]. Secondly, soil animals improve the utilization efficiency of soil nutrients by directly participating in the primary process of material circulation. For example, soil invertebrates can intensely break down and decompose soil organic matter, thus accelerating the mineralization process of organic matter and further leading to the decrease in TC content in soil [33]. Thirdly, high levels of N additions may exacerbate the soil carbon leaching process, leading to a potential decrease in TC content [32].

N addition inhibits organic phosphorus mineralization and reduces inorganic phosphorus levels in the soil, further reducing phosphorus uptake and utilization by plants and microorganisms. These results may be caused by soil acidification [31,34]. After interruption of short-term N additions in our study, soil acidification was mitigated, and the rate of organic phosphorus mineralization was increased, which further enhanced phosphorus utilization by plants and microorganisms and ultimately led to a decrease in TP content. Previous studies have demonstrated that N additions can alleviate N limitation in *Larix olgensis* forests, promote soil nutrient cycling, and significantly increase the abundance of beneficial microbial taxa in the soil [25]. These findings indicate that the impact of N addition on the soil is lagging, and the impacts of high N addition on TP content are more intense than those of low N addition. Plants and microorganisms exhibit a higher demand for phosphorus, which could explain the lower levels of TP content at high N additions. 

Soil stoichiometric ratios are extensively used to investigate nutrient balances in soil ecosystems and the response of biogeochemical cycles to external nutrient element inputs [35]. Previous studies have showed that long-term persistent N addition affects soil microbial community composition and enzyme activity by altering soil C:P and N:P ratios [36,37]. Forest soils with high C:P ratio promote bacterial diversity [38]. The soil C:N ratio after interruption ranged from 10.5 to 13.5, which was basically within the range of the carbon–nitrogen ratio of wet temperate soil (10–12) in China. When the soil C:N ratio is greater than 10, N application can, to some extent, alleviate the limitation of nitrogen on soil microorganisms [16]. Soil C:N:P ratios were also lower than the global ratio of 186:13:1 (about 14.3) in soil [37]. After interruption of short-term N additions, N addition significantly affected TC, TN, and TP contents. Except the C:N ratio, other stoichiometric ratios increased significantly after interruption. This implied that although N addition affected soil elemental balances, their positive effects on soil nutrients persisted after interruption and drove soil change in a healthier direction.

### 4.2. Changes in Soil Bacterial Diversity before and after Interruption of Short-Term Nitrogen Additions

The changes in the number of bacterial OUTs visualized the impact of N addition on the number of bacterial communities in each treatment. The bacterial diversity index did not change much in 2023, but the bacterial richness index decreased significantly (*p* < 0.01). Studies have shown that N addition can alleviate soil nitrogen deficiency and increase bacterial diversity by changing soil availability [25]. In general, the decrease in Chao1 index after N addition is greater than that of Shannon index in most studies. The greater the decrease in Chao1 index, the more rare species disappear in the soil [16]. N addition increased the bacterial Shannon index, which indicated that the evenness of soil bacteria increased, but the bacterial Chao1 index still decreased possibly due to a decrease in rare species under N addition [16,39], which is consistent with our findings. N addition and precipitation affect microbial communities by altering soil pH; N addition decreases pH, while precipitation increases pH [40]. Correlation analysis showed that bacterial Chao1 index decreased significantly with increasing water content (*p* < 0.05). During the same period, August received more rainfall than 2021 in the study area.

### 4.3. Changes in Soil Bacterial Structure at the Phylum Level before and after Interruption of Short-Term Nitrogen Additions

Compared to pre-interruption, the community dominant flora were essentially the same before and after interruption, but the average relative abundance varied. This indicates that some phylums of soil microorganisms have certain adaptability [41,42,43]. And the relative abundance of *Acidobacteriota*, *Actinobacteriota*, *Chloroflexi*, *Verrucomicrobiota*, and *Planctomycetota* all increased, and the relative abundance of *Actinobacteriota* increased the most, regardless of previous levels of N addition. However, the relative abundances of *Myxococcota* and *Proteobacteria* were decreased, and the relative abundance of *Proteobacteria* decreased the most.

Studies have shown that *Actinomycetes* can promote microbial production of more lignocellulose hydrolases, thereby promoting the degradation of organic matter [33,44]. *Acidobacteriota* are often important contributors to the nutrient cycling system in soil microorganisms and are more likely to settle in soils with lower pH values [45]. *Firmicutes*, *Chlorobacteria*, and *Verrucomicrobia* are important participants in the carbon and nitrogen cycles, and their activity is inhibited by high N addition [46]. Different studies have shown that *Planctomyces* is a typical microbial group involved in the soil nitrogen cycle. *Proteobacteria* is more sensitive to soil pH and tends to colonize in soil with lower pH; the increase in soil pH value after interruption may be the most critical reason for the decrease in the relative abundance of *Proteobacteria* [47]. *Myxococcota* is considered to have a critical role in the turnover of biochar and other unstable organic matter in soil ecosystems [48]. Soil organic matter produces acid and amide compounds during decomposition, and *Methylomirabilota* can accelerate the decomposition of organic matter by promoting the synthesis of organic acid compounds [49,50]. The increase in the relative abundance of *Methylomirabilota* indicates that the interruption after short-term N additions promotes soil carbon cycling. *Nitrospirota* are sensitive to changes in soil nutrients [51]. Our result shows that the relative abundance of *Nitrospirota* decreased, and although the relative abundance was less than 1%, the decrease in *Nitrospirae* showed that nitrification activity might decline under interruption after short-term N additions.

### 4.4. Changes in Soil Bacterial Structure at the Genus Level before and after Interruption of Short-Term Nitrogen Additions

Compared to 2021, *Gemmatimonas*, *Sphingomonas*, and *Pseudolabrys* remained among the dominant taxa with high relative abundance. In addition, this study also identified the relatively high abundance of beneficial flora *Ellin6067*, *Massilia*, *Solirubrobacter*, and *Bradyrhizobium*. *Gemmatimonas* is generally abundant in low-moisture soils, which can participate in the decomposition of soil organic matter and contribute to the soil carbon cycle [52,53]. Previous studies have shown that *Sphingomonas* and *Massilia* have a strong ability to degrade polycyclic aromatic hydrocarbons in soil, which is conducive to soil ecological restoration [54]. In addition, *Sphingomonas* can fix nitrogen in the soil. *Massilia* is identified as a key taxon for enhancing microbial community resilience under unfavorable conditions [55]. *Pseudomonas* and *Ellin6067* belong to *Proteobacteria*; the increase in *Pseudomonas* is beneficial for soil nitrogen fixation [56], while *Ellin6067* is associated with ammonia oxidation, which is conducive to promoting soil nitrification [57]. *Solirubrobacter* belongs to *Actinobacteriota*, which is associated with the mineralization of soil organic matter and the release of available nutrients to plants. Thus, the increase in the relative number of *Solirubrobacter* indicates the improvement of soil fertility and productivity [58,59]. Previous studies have indicated that the increase in the relative abundance of the dominant nitrogen-fixing bacterium *Bradyrhizobium* may be regulated by soil phosphorus, and the increase in TP content under interruption may be the reason for the increase in its relative abundance [60,61].

## 5. Conclusions

Based on the above research, interruption after short-term N addition had a positive impact on soil nutrient cycling in *Larix olgensis* forests. Soil C:P and C:N:P increased significantly, indicating that soil nitrogen and phosphorus limitations were alleviated. Compared with the pre-interruption period, the bacterial richness index decreased after interruption, but the bacterial diversity index increased, and the relative abundance of beneficial microbial taxa increased. Interruption after short-term nitrogen additions favored soil health conditions and microbial growth in the *Larix olgensis* forest. The main environmental factor affecting the microbial richness index was SWC, and the primary environmental factors affecting the structure of soil microbial communities were TP and SWC. This indicates that SWC and TP may be the main reasons for the changes in soil bacterial community diversity and structure in research sites after interruption. In general, interruption after short-term nitrogen additions contributes to the ecological stability of *Larix olgensis* forest soil. The microbial dynamics under nitrogen saturation should be studied for an extended period of time to pay more attention to the functional dynamics of the microorganisms in the future and to further explore the possible conditions for a more stabilized soil ecology. These experimental results will provide support for further investigation of the short-term response of soil microbial community structures to N deposition and its self-regulation ability in the forest soils of northern China against the global climate change background. 

## Figures and Tables

**Table 1:** Analysis of interruption after short-term N additions on soil environmental factors and Soil microbial diversity.

Treatments	Nitrogen Addition		Interruption		Nitrogen Addition × Interruption	
	F	*p*	F	*p*	F	*p*
Soil pH	18.328	0.001 **	18.328	0.000 **	1.251	0.310
SWC (%)	6.695	0.006 **	1.180	0.292	6.843	0.006 **
TC (mg/kg)	115.241	0.000 **	0.136	0.736	2.135	0.147
TN (mg/kg)	229.710	0.000 **	5.396	0.030 *	26.614	0.000 **
TP (mg/kg)	20.908	0.001 **	13.428	0.001 **	13.808	0.000 **
C:N	287.558	0.000 **	15.851	0.001**	7.392	0.005 **
C:P	61.143	0.000 **	436.935	0.000 **	10.140	0.001 **
N:P	150.122	0.000 **	26.264	0.000 **	54.458	0.000 **
C:N:P	209.255	0.000 **	291.396	0.001 **	12.913	0.000 **
Chao1	4.768	0.018 *	2049.800	0.001 **	7.026	0.004 **
Shannon	8.839	0.001 **	5.952	0.752	10.660	0.000 **
Simpson	6.484	0.006 **	16.658	0.000 **	3.213	0.058

* Indicates significant correlation at the 0.05 level, ** indicates high correlation at the 0.01 level.

## Data Availability

The raw data supporting the conclusions of this article will be made available by the authors on request.
